# Production of the Neurotoxin Salsolinol by a Gut-Associated Bacterium and Its Modulation by Alcohol

**DOI:** 10.3389/fmicb.2018.03092

**Published:** 2018-12-18

**Authors:** Daniel N. Villageliú, David J. Borts, Mark Lyte

**Affiliations:** ^1^Department of Veterinary Microbiology and Preventive Medicine, College of Veterinary Medicine, Iowa State University, Ames, IA, United States; ^2^Interdepartmental Microbiology Graduate Program, College of Veterinary Medicine, Iowa State University, Ames, IA, United States; ^3^Department of Veterinary Diagnostic and Production Animal Medicine, College of Veterinary Medicine, Iowa State University, Ames, IA, United States

**Keywords:** salsolinol, Parkinson’s disease, microbial metabolic activity, gut-brain-axis communication, gut origin for Parkinson’s disease

## Abstract

Utilizing a simulated gastrointestinal medium which approximates physiological conditions within the mammalian GI tract, experiments aimed at isolating and identifying unique microbial metabolites were conducted. These efforts led to the finding that *Escherichia coli*, a common member of the gut microbiota, is capable of producing significant quantities of salsolinol. Salsolinol is a neuroactive compound which has been investigated as a potential contributor to the development of neurodegenerative diseases such as Parkinson’s disease (PD). However the origin of salsolinol within the body has remained highly contested. We herein report the first demonstration that salsolinol can be made *in vitro* in response to microbial activity. We detail the isolation and identification of salsolinol produced by *E. coli*, which is capable of producing salsolinol in the presence of dopamine with production enhanced in the presence of alcohol. That this discovery was found in a medium that approximates gut conditions suggests that microbial salsolinol production could exist in the gut. This discovery lays the ground work for follow up *in vivo* investigations to explore whether salsolinol production is a mechanism by which the microbiota may influence the host. As salsolinol has been implicated in the pathogenesis of PD, this work may be relevant, for example, to investigators who have suggested that the development of PD may have a gut origin. This report suggests, but does not establish, an alternative microbiota-based mechanism to explain how the gut may play a critical role in the development of PD as well other conditions involving altered neuronal function due to salsolinol-induced neurotoxicity.

## Introduction

**FIGURE 1:**
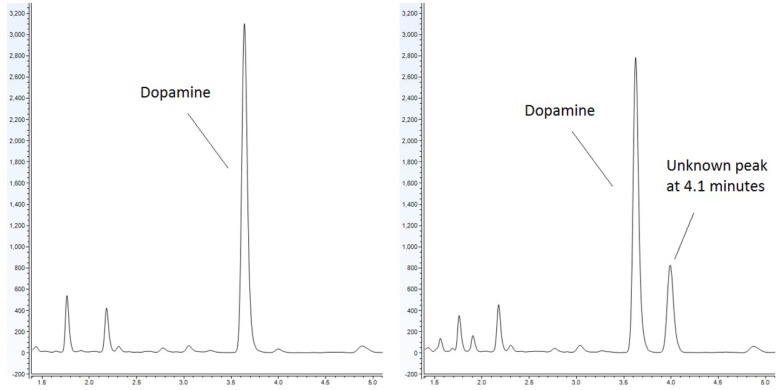
Response of *E. coli* to dopamine analyzed by UHPLC-ECD. **(Left)** Control (non-inoculated) simulated small intestinal medium supplemented with 1 mM dopamine. **(Right**) Dopamine supplemented medium with organisms grown within generates a distinct chromatographic signal indicative of the production of a new chemical. This effect is not observed when *E. coli* is grown in the absence of dopamine.

Emerging evidence has suggested that there is an association between certain diseases and an altered gut-microbiome. Metabolites produced in the gut have the capacity to modulate disease (Lyte, 2016). It has been noted that bacteria produce and utilize many of the same neurochemicals that are used by the host’s neurophysiological system with clinical implications ranging from infection to behavioral modification through the microbiota-gut-brain axis (Lyte, 2014; Lyte and Brown, 2018). Our lab has sought to identify metabolites, which are produced by the microbiota and have the capacity to influence the host. As part of our approach, we utilize a food-based simulated small intestinal medium (sSIM) which simulates the gastrointestinal environment following food consumption. This enabled us to report the ability of common bacterial genera present within the gut to produce neurochemicals that otherwise would not be detected in more common laboratory media [Luria-Bertani broth (LB) as a prototypical example] which do not accurately reflect the gut *in vivo* milieu (Villageliu et al., 2018). During these experiments we noted the appearance of a prominent microbial product of unknown identity (Figure 1) when cultures were inoculated with *Escherichia coli*. As detailed herein, we determined that this microbial product was salsolinol.

One of the most investigated chemicals implicated in the development of PD is salsolinol, a chemical which was first associated with PD in the 1970s when it was detected in the urine of patients receiving L-dopa therapy (Kurnik-Łucka et al., 2018). Pharmacologically, salsolinol is a tetrahydroisoquinoline derivative with neuroactive properties most often viewed in the context of its potential as a neurotoxin (Kang, 2013; Możdżeń et al., 2015; Chen et al., 2018). Possessing the group defining catechol moiety of catecholamines, salsolinol can interact with various adrenergic receptors. Additionally, it has been shown that (R)-SAL and (S)-SAL activate the μ-opioid receptor by the classical G protein-adenylate cyclase pathway in a morphine-like interaction (Berríos-Cárcamo et al., 2016). There is some evidence that salsolinol may cross the blood brain barrier. *In vivo*, intraperitoneal administration resulted in accumulation of salsolinol within the neostriatum in dialysate (Quintanilla et al., 2014). It is known that other tetrahydroisoquinolone derivatives can cross the blood–brain barrier and it has also been reported that salsolinol administered systemically, can alter laboratory animal behavior which indirectly suggests that salsolinol could cross the blood-brain barrier (Kurnik-Łucka et al., 2018).

Salsolinol may lead to oxidative stresses that culminate in the protein aggregation of alpha-synuclein, an activity highly relevant in PD. Salsolinol induces an oxidative modification in cytochrome c. When cytochrome c is incubated with salsolinol, cytochrome c is oxidized and aggregates in a concentration dependent manner (Kang, 2013). Cytochrome c may co-localize with alpha-synuclein and initiate the oligomerization of alpha-synuclein (Kumar et al., 2016) and it is conceivable that cytochrome c altered by salsolinol may trigger or enhance this effect. More generally, salsolinol may be a neurotoxic and oxidative insult that could initiate a cascade leading or contributing to disease.

To date, the routes through which salsolinol is produced and accumulates within the body, particularly within the brain, have been contested. The presence of salsolinol within the environment has long been recognized and the synthesis of simple tetrahydroisoquinolines under mild physiological conditions such as those that occur in plants was described as far back as 1936 (Georg and Stiehl, 1936). Many dietary sources of salsolinol have been reported, with some sources like bananas having in excess of 5 μg salsolinol/gram wet weight (Kurnik-Łucka et al., 2018). An unequal distribution in the R vs. S enantiomers within the brain has led some to suggest the possibility of *in situ* synthesis by a “salsolinol synthase” which selectively forms the R enantiomer. Within the last year, a salsolinol synthase enzyme has been identified and described (Chen et al., 2018). Conversely, it has also been shown that salsolinol can be formed by the Pictet-Spengler mechanism in which aldehyde and dopamine react to form a racemic mixture of both the R and S forms of salsolinol (Kurnik-Łucka et al., 2018).

The finding that enterobacteria can produce salsolinol is significant. In addition to offering a possible explanation as to where salsolinol may be generated with the body, the production of salsolinol suggests a novel mechanism by which the microbiota might impact host health. There are many examples of the microbiota modulating inflammatory conditions, and proteobacteria (of which enterobacteriacae is a family) are commonly associated with human disease (Rizzatti et al., 2017). The finding that enterobacteriacae can produce salsolinol corroborates studies which show that metabolites derived from the microbiota may impact neurological conditions as well. For example, in a study which utilized microbiome transplants from healthy and Parkinsonian human donors into mice which overexpressed the alpha-synuclein protein, it was demonstrated that mice with a microbiota from Parkinson’s patients increased impairment (Sampson et al., 2016). In particular, it has recently been recognized that enterobacteriaceae are positively associated with the severity of postural instability and gait difficulty in Parkinson’s disease (Scheperjans et al., 2015; Tremlett et al., 2017).

While we have chosen to detail the significance of salsolinol to Parkinson’s disease as a prototypic example, it should also be noted that the production of salsolinol carries implications beyond those of Parkinson’s disease. For example, the ability of salsolinol to modulate the action of ethanol on neuronal activity in the region of the brain associated with motivation has been shown to occur in dopaminergic neurons (Melis et al., 2015). It has been suggested that an opioid action of salsolinol or its enantiomers is involved in the rewarding effects of ethanol (Berríos-Cárcamo et al., 2016).

## Materials and Methods

### Microorganisms

Twelve isolates of enterobacteria were obtained. Of these twelve, eight of these were isolates of *E. coli*: four were environmental isolates from livestock including chickens (ML1160-ML1162) and swine (ML1084); strains designated BW25113 and JW1228 were obtained from the Coli Genetic Stock Center (New Haven, CT, United States) and represent the parent strain of the Keio knock collection and and alcohol dehydrogenase mutant, respectively (Baba et al., 2006). Isolates of *Enterobacter cloacae* and *Citrobacter freundii* were obtained from the Iowa State Veterinary Medicine Diagnostic Laboratory.

### Chemicals and Reagents

Salsolinol hydrobromide was purchased from Sigma-Aldrich (St. Louis, MO, United States). LC-MS grade acetonitrile, methanol, water, formic acid, and ammonium acetate were purchased from Fisher Scientific (Pittsburgh, PA, United States).

### Processing of Samples for HPLC With Electrochemical Detection

Optimized conditions for the processing, separation and quantification of catecholamines from bacterial cultures were previously developed (Villageliu et al., 2018). Post growth, cultures were acidified with the addition of 10 μL of 10N hydrochloric acid (HCl) for every 1 mL of medium. Culture medium was centrifuged (3000 × *g*, 4°C for 15 min) to remove insoluble fiber, denatured proteins and other precipitates. The sample supernatant was further purified by passage through a 2 kDa molecular weight cut off filter (MWCO). Samples were stored at -80°C.

Quantification of neurochemicals was performed by UHPLC-ECD on a Dionex UHPLC system which consisted of the following components: a Dionex Ultimate 3000 autosampler, a Dionex Ultimate 3000 pump and a Dionex Ultimate 3000 RS electrochemical detector (Thermo Scientific, Sunnyvale, CA, United States). Separation was achieved using buffered 10% acetonitrile mobile phase (MD-TM mobile phase, Thermo Scientific), a 150 mm, 3 μm Hypersil BDS C18 column (Thermo Scientific) and flow rate of 0.6 mL min^-1^. Prior to injection, samples were held at 4°C by the autosampler. Electrochemical detection (ECD) was achieved with a 6041RS glassy carbon electrode set to 400 mV.

### Isolation of Salsolinol

*Escherichia coli* was grown anaerobically in sSIM (Villageliu et al., 2018) for 24 h in the presence of 1 mM dopamine. Following growth, samples were acidified with the addition of 10 μL of 10 M HCl for every 1 mL of medium. To ensure acidification did not contribute to salsolinol formation, a second subset of samples was processed without acid treatment. Samples were centrifuged at 3000 × *g* for 15 min at 4°C. Supernatant was passed through a 2 kDa molecular weight cut off filter and analyzed by UHPLC-ECD. Chromatography demonstrated the presence of a distinct chemical response with a retention time of 4.1 min in both acidified and centrifuge only subsets. 5 mL of filtered supernatant was further treated by mixing with 400 mg of affinity beads specific for catechols (Bio-Rad, Hercules, CA, United States). UHPLC was done on eluted samples to ensure the presence of the peak at 4.1 min. Fractional collection based on retention time was used to isolate the peak from remaining impurities. Fractions were pooled and concentrated by centrifugal rotoevaporation using a (CentriVap^®^ Labconco, Kansas City, MO, United States). Concentrated samples were reanalyzed by HPLC-ECD to ensure that no deviation in retention time had occurred before delivery of the sample for analysis by LC-MS. Upon identification of the peak as salsolinol, a salsolinol standard (Sigma-Aldrich) was ordered and tested on UHPLC-ECD. The retention time of this standard was an identical match.

### LC-MS Analysis

LC-MS analyses were performed using an Ultimate 3000 UHPLC system coupled to a Q Exactive Focus hybrid quadrupole-orbitrap mass spectrometer (Thermo Fisher Scientific, San Jose, CA, United States).

For reversed phase chromatography, the LC column was an Accucore aQ, 2.6 μm, 2.1 × 100 mm from Thermo Fisher Scientific (Waltham, MA, United States). The mobile phase consisted of (A) 0.1% formic acid in water and (B) 0.1% formic acid in methanol. The LC gradient was: start to 1.0 min: 0% B, linear ramp to 11.0 min: 98% B, held for 2 min, and then returned to initial conditions and held for 2 min prior to the next injection. The flow rate was 300 μL/min and the column temperature was maintained at 35°C. An injection volume of 10 μL was used.

For HILIC (hydrophilic interaction liquid chromatography), the LC column was a SeQuant ZIC-HILIC, 5 μm, 200 Å, 2.1 × 150 mm from Millipore Sigma (Darmstadt, Germany). The mobile phase consisted of (A) 95/5 (v/v) water/acetonitrile containing 10 mM ammonium acetate and (B) 5/95 (v/v) water/acetonitrile containing 10 mM ammonium acetate. The LC gradient was: start to 0.5 min: 99% B, linear ramp to 15.5 min: 50% B, held for 3 min, returned to initial conditions over 1 min, and held for 8 min prior to the next injection. The flow rate was 500 μL/min and the column temperature was maintained at 40°C. An injection volume of 10 μL was used in full mass range mode and a 1 μL injection volume was used in MS/MS mode.

For LC-MS analysis with reversed phase chromatography, the mass spectrometer was operated in positive and negative electrospray ionization (ESI) mode. In positive ESI mode the following conditions were used: spray voltage: 3.5 kV, ion transfer capillary temperature: 256°C, S-lens RF level: 55, sheath gas flow rate: 48, auxiliary gas flow rate: 11, and sweep gas flow rate: 2. In negative ESI mode, the following conditions were used: spray voltage: 2.5 kV, ion transfer capillary temperature: 256°C, S-lens RF level: 55, sheath gas flow rate: 48, auxiliary gas flow rate: 11, and sweep gas flow rate: 2. (S-lens RF level and gas flow rates are in arbitrary units.) For full mass range analysis, the orbitrap was operated at a resolution setting of 70,000 over a *m/z* range of 100–1000 with an automatic gain control setting of 1E10^6^ and a maximum ion time setting of auto. For MS/MS analysis, the mass spectrometer was operated in parallel reaction monitoring (PRM) mode at a resolution of 35,000. Collision energy settings of 20, 30, or 40 were used in both positive and negative ESI mode.

For LC-MS analysis with HILIC chromatography, the mass spectrometer was operated in positive electrospray ionization (ESI) mode using the following conditions: spray voltage: 3.0 kV, ion transfer capillary temperature: 350°C, S-lens RF level: 45, sheath gas flow rate: 60, auxiliary gas flow rate: 15, and sweep gas flow rate: 2. (S-lens RF level and gas flow rates are in arbitrary units.) For full mass range analysis, the orbitrap was operated at a resolution setting of 70,000 over a mass range of 65–975 with an automatic gain control setting of 1E10^6^ and a maximum ion time setting of auto. For MS/MS analysis, the mass spectrometer was operated in parallel reaction monitoring (PRM) mode at a resolution of 35,000 and collision energy settings of 20, 30, or 40.

Data were acquired using Xcalibur 4.0 software and product ion spectra were searched against the mzCloud online mass spectral database^1^ using FreeStyle 1.1 software (all from Thermo Fisher Scientific, San Jose, CA, United States).

**FIGURE 2:**
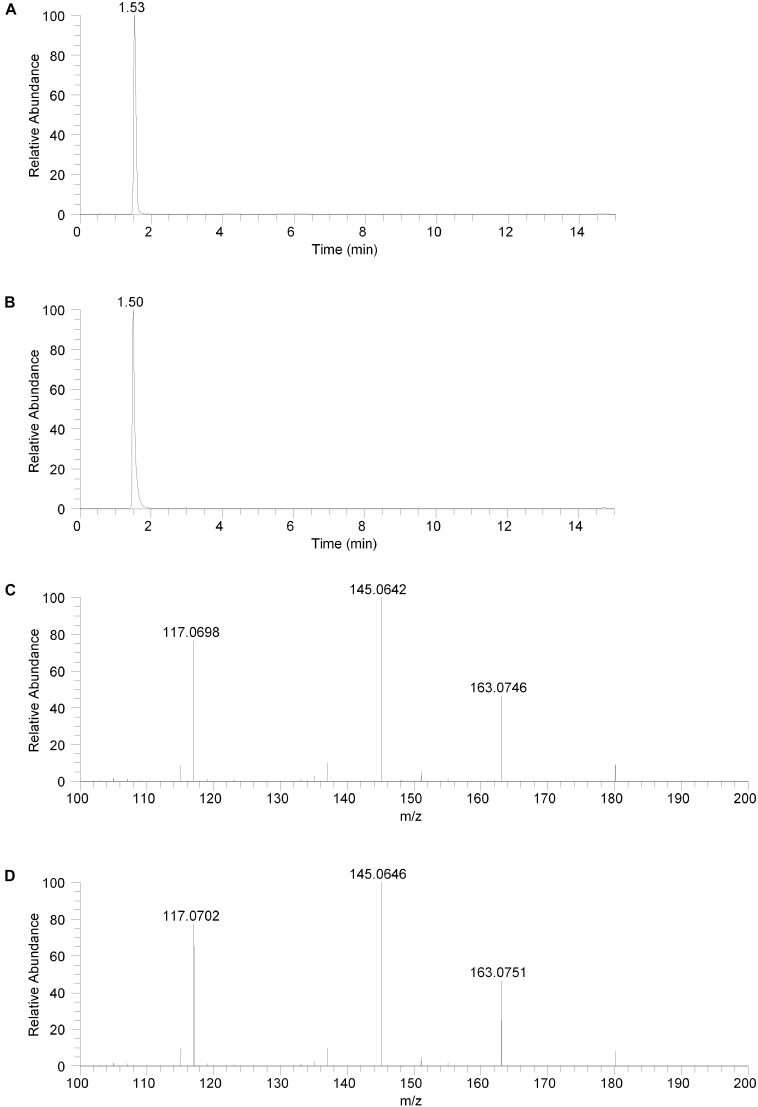
Reversed phase chromatography positive ion electrospray extracted ion chromatograms and product ion spectra. **(A)** Extracted ion chromatogram for the *m/z* 180 ion in the isolated fraction. **(B)** Extracted ion chromatogram for the *m/z* 180 ion from the salsolinol analytical standard. **(C)** Product ion spectrum (CE setting = 20) for the *m/z* 180 ion in the isolated fraction. **(D)** Product ion spectrum (CE setting = 20) for the *m/z* 180 ion in the salsolinol analytical standard.

The isolated fraction was initially analyzed using reversed phase LC-MS in positive ion ESI mode with full mass range analysis. The total ion chromatogram (TIC) revealed significant peaks at retention times of 0.66 and 0.95 min and a smaller peak at a retention time of 1.53 min. Inspection of the mass spectrum of the peaks at 0.66 and 0.95 min indicated that these peaks were consistent with salt and solvent clusters. Inspection of the mass spectrum of the peak at 1.53 showed an ion with a measured accurate *m/z* of 180.1015. An extracted ion chromatogram (XIC) for the *m/z* 180.1015 ion (±2.5 ppm) is shown in Figure 2A. Product ion spectra were acquired on the *m/z* 180.1015 ion at collision energy (CE) settings of 20, 30, and 40. The product ion spectrum for the *m/z* 180.1015 ion at CE = 20 is shown in Figure 2C. FreeStyle 1.1 software was used to process this spectrum and submit it for database searching against the mzCloud^1^ online orbitrap-based product ion spectral database. (The mzCloud database contains data on over 8000 small molecule compounds and has nearly 3 million spectra). The top database search result returned was for the compound salsolinol. The modified NIST match score for salsolinol was 92.9. The compound with the next closest modified NIST match had a score of 78.2. Salsolinol was also the top database search result, with similar match scores, for the product ion spectra acquired at CE = 30 and 40. In addition, the exact *m/z* measured for the compound, 180.1015, was consistent with the calculated exact *m/z* for salsolinol, 180.1019, with 2.2 ppm error. All of these results are consistent with the unknown compound in the isolated fraction corresponding to salsolinol.

The isolated fraction was next analyzed using reversed phase LC-MS in negative ion ESI mode with full mass range analysis. The TIC revealed a significant peak at a retention time of 0.66 min and a smaller peak at a retention time of 1.53 min. Inspection of the mass spectrum of the peak at 0.66 min indicated that this peak was consistent with salt and solvent clusters. Inspection of the mass spectrum of the peak at 1.53 showed an ion with a measured accurate *m/z* of 178.0862. Product ion spectra were acquired on the *m/z* 178.0862 ion at collision energy (CE) settings of 20, 30, and 40 (data not shown). FreeStyle 1.1 software was used to process the product ion spectrum collected with a CE setting of 20 and to submit it for database searching against the mzCloud^1^ online orbitrap-based product ion spectral database. The top database search result returned was for the compound salsolinol. The modified NIST match score for salsolinol was 93.8. The compound with the next closest modified NIST match had a score of 36.6. Salsolinol was also the top database search result, with similar match scores, for the product ion spectra acquired at CE = 30 and 40. In addition, the exact *m/z* measured for the compound, 178.0862, was consistent with the calculated exact *m/z* for salsolinol, 178.0873, with 6.2 ppm error. All of these results are also consistent with the unknown compound in the isolated fraction corresponding to salsolinol.

To confirm the putative identification of the compound contained in the fraction, an analytical standard of salsolinol was acquired and analyzed using reversed phase LC-MS positive ion ESI mass spectrometry procedures identical to the unknown fraction. The salsolinol analytical standard material showed a retention time of 1.50 min with the reversed phase LC-MS system. This slight difference in retention time compared with the compound in the isolated fraction (1.53 min) is likely due to the high salt content in the isolated fraction. The measured exact *m/z* for the salsolinol analytical standard was 180.1019, which matches the calculated exact mass of 180.1019 with 0.0 ppm error. An XIC for the *m/z* 180.1019 ion is shown in Figure 2B. Product ion spectra for the salsolinol analytical standard were acquired at CE settings for 20, 30, and 40. The spectra at each of these CE settings were virtually identical to the compound in the isolated fraction. The product ion spectrum for the salsolinol analytical standard at CE = 20 is shown in Figure 2D.

To further confirm the identity of the compound in the isolated fraction as salsolinol, both the isolated fraction and the analytical standard salsolinol material were analyzed using an orthogonal chromatography mode known at hydrophilic interaction liquid chromatography or HILIC. In HILIC, polar compounds are generally more highly retained and elute later while more non-polar compounds are generally less highly retained and elute earlier. This is the reverse of the elution order with reversed phase liquid chromatography (McCalley, 2017).

**FIGURE 3:**
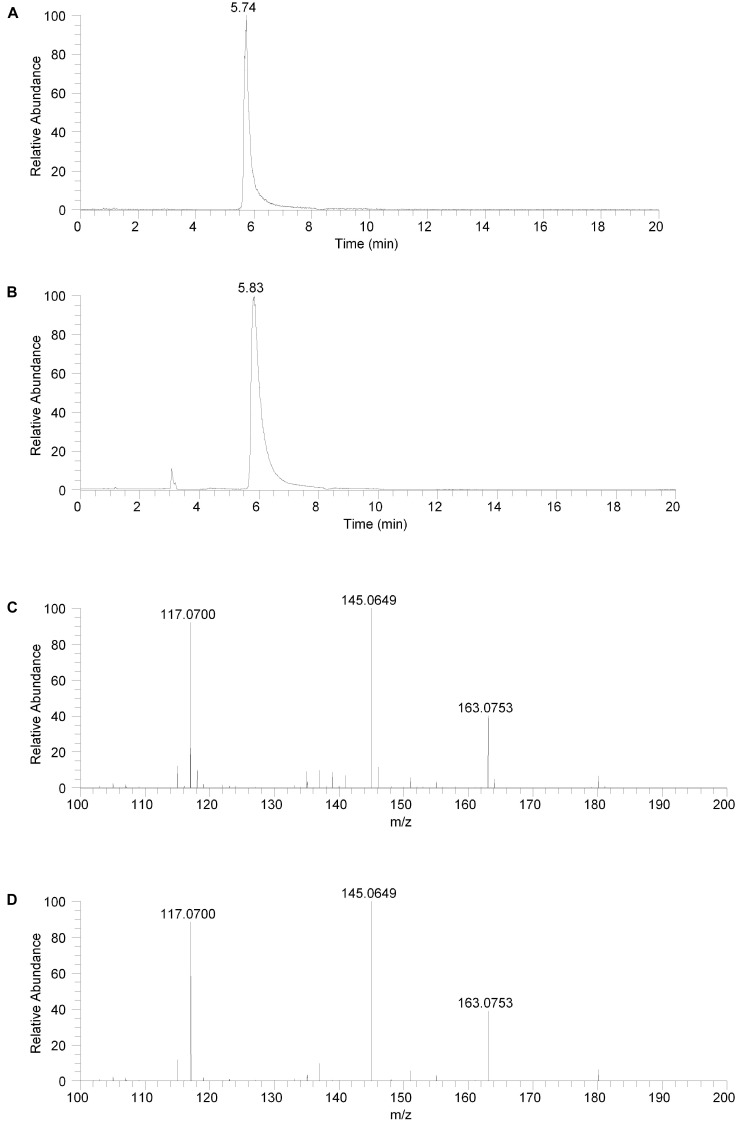
Hydrophilic interaction liquid chromatography (HILIC) positive ion electrospray extracted ion chromatograms and product ion spectra. **(A)** Extracted ion chromatogram for the *m/z* 180 ion in the isolated fraction. **(B)** Extracted ion chromatogram for the *m/z* 180 ion from the salsolinol analytical standard. **(C)** Product ion spectrum (CE setting = 20) for the *m/z* 180 ion in the isolated fraction. **(D)** Product ion spectrum (CE setting = 20) for the *m/z* 180 ion in the salsolinol analytical standard.

The isolated fraction and salsolinol analytical standard were analyzed using HILIC LC-MS in positive ion ESI mode with full mass range analysis and MS/MS analysis. The XICs for *m/z* 180.1019 (± 2.5 ppm) for the isolated fraction and salsolinol analytical standard are shown in Figures 3A,B, respectively. Again, we believe the slight difference in retention times, 5.74 min for the isolated fraction versus 5.83 min for the analytical standard, can be attributed to the high salt content in the isolated fraction. The measured accurate mass for the *m/z* 180 component in the isolated fraction was 180.1020 while the measured accurate mass for the salsolinol analytical standard was 180.1019. These measured masses have 0.6 and 0.0 ppm error compared to the calculated exact *m/z* for salsolinol and 0.6 ppm error compared to one another.

Product ion spectra for the *m/z* 180 component in the isolated fraction and the salsolinol analytical standard were acquired in HILIC LC-MS positive ion ESI mode at CE settings of 20, 30, and 40. For all three CE settings, the product ion spectra for the *m/z* 180 component of the isolated fraction and the salsolinol analytical standard were virtually identical. The product ion spectra at CE settings of 20 for the *m/z* 180 component in the isolated fraction and the salsolinol analytical standard are shown in Figures 3C,D, respectively. Upon identification of molecule as salsolinol, a salsolinol standard was ordered and tested on UHPLC-ECD. The retention time of this standard was an identical match to the original unknown peak.

### Experimental Culture Conditions

Isolates used for inoculation were grown on TSA agar with 5% ovine blood overnight. Harvested colonies were suspended in peptone water and standardized to an OD_600_ of 0.2. Inoculation was achieved by mixing 100 μL of organism suspension with 5 mL of medium. The production of salsolinol by various species of *Enterobacteriaceae* was tested in five types of media: Luria broth (LB), De Man, Rogosa, Sharpe (MRS) broth, brain heart infusion broth (BHI), tryptic soy broth (TSB), and sSIM. All media were tested and found to have little or no dopamine in their native state. Dopamine was supplemented by adding 100 μL of a 0.2 μm filtered 0.05 M solution of dopamine hydrochloride to a total volume of 5 mL of medium.

Culture broths were grown anaerobically, at 37°C for 24 h. In the case of sSIM, which is a complex suspension, agitation was provided with magnetic stir bars to keep particles evenly distributed. After 24 h, samples were processed for analysis by UHPLC.

## Results

*Escherichia coli* was grown anaerobically in sSIM for 24 h in the presence of 1 mM dopamine. Following growth, samples were acidified with the addition of 10 μL of 10M HCl for every 1 mL of medium. To ensure acidification did not contribute to salsolinol formation, a second subset of media samples was processed without acid treatment. Samples were centrifuged at 3000 × g for 15 min at 4°C. Supernatant was passed through a 2 kDa molecular weight cut off filter and analyzed by UHPLC (ultra-high performance liquid chromatography)-ECD (electrochemical detection). UHPLC-ECD demonstrated the presence of a distinct chemical response with a retention time of 4.1 min in both acidified and centrifuge only subsets (Figure 1).

5 mL of filtered supernatant was further treated by mixing with 400 mg of affinity beads specific for catechols (Bio-Rad, Hercules, CA, United States). UHPLC was performed on eluted samples to ensure the presence of the peak at 4.1 min remained. Fractional collection based on retention time was used to isolate and further purify the peak. Fractions were pooled and concentrated by a centrifugal concentrator (Labconco, Kansas City, MO, United States) and then subjected to analysis by LC-MS (liquid chromatography-mass spectrometry) for identification of the collected peak.

The compound in the isolated fraction was identified and confirmed as salsolinol by analysis in full mass range and tandem mass spectrometry (MS/MS) modes at multiple collision energies using a UHPLC LC-MS system (Thermo Fisher Scientific, San Jose, CA, United States). Results from these analyses were consistent with those obtained using an authentic analytical standard of salsolinol. Chromatographically, both reversed-phase and orthogonal HILIC (hydrophilic interaction liquid chromatography) separation modes were employed. In all cases, the chromatographic retention times matched between the compound in the isolated fraction and the salsolinol standard. In addition, the measured accurate masses (*m/z* ratios) for all pseudomolecular [(M+H)^+^ and (M-H)^-^] ions and product ions were consistent between the compound in the isolated fraction and the salsolinol standard. The measured accurate masses were also consistent with calculated exact masses within expected experimental error. Product ion relative intensities were consistent between the compound in the isolated fraction and the salsolinol standard at all collision energies. Extracted ion chromatograms and product ion spectra for the compound in the isolated fraction and the salsolinol analytical standard are shown in Figures 2, 3. Following the assignment of the isolated fraction as salsolinol by mass spectrometry, we were able to match the retention time of a commercial salsolinol standard (Sigma-Aldrich, St. Louis, MO, United States) to that of experimental samples.

Medium choice appears to play a significant impact on the final concentration of salsolinol observed. Twelve isolates of enterobacteria were obtained. Of these twelve, eight of these were isolates of *E. coli*: four were environmental isolates from livestock including chickens (ML1160-ML1162) and swine (ML1084); strains designated BW25113 and JW1228 were obtained from the Coli Genetic Stock Center and represent the parent strain of the Keio knock out collection and an alcohol dehydrogenase mutant, respectively (Baba et al., 2006). Isolates of *Enterobacter cloacae* and *Citrobacter freundii* were obtained from the Iowa State Veterinary Medicine Diagnostic Laboratory.

In 8 of the 10 trials done with enterobacteria, the greatest amount of salsolinol was produced in MRS, averaging 220 μM among *E. coli* strains. In un-inoculated controls supplemented with 1 mM dopamine, only trace salsolinol was detected. Production in MRS showed comparatively little variation, with the standard deviation of the mean averaging 6.6 μM across six *E. coli* strains. Production of salsolinol in sSIM was also robust, averaging 174 μM across the *E. coli* strains. Production in BHI and TSB was relatively limited, averaging 90 μM in BHI and only 76 μM in TSB. For *E. coli*, there was no discernable production of salsolinol in LB and salsolinol levels averaging 7 μM were comparable to the LB controls. The addition of ethanol substantially impacted the salsolinol production of five tested enterobacteria. The effect was particularly noticeable in *E. coli* from the Keio knock out collection in which the wild type generated an average of 206 μM (±8 μM) in the absence of alcohol, but a remarkable 920 μM (±8 μM) in the presence of 4% ethanol. This production was essentially unaffected in the single alcohol dehydrogenase knock out JW1228 which generated an average of 232 μM (±16 μM) in the absence of alcohol and 890 μM (±28 μM) in the presence of ethanol.

## Discussion

**FIGURE 4:**
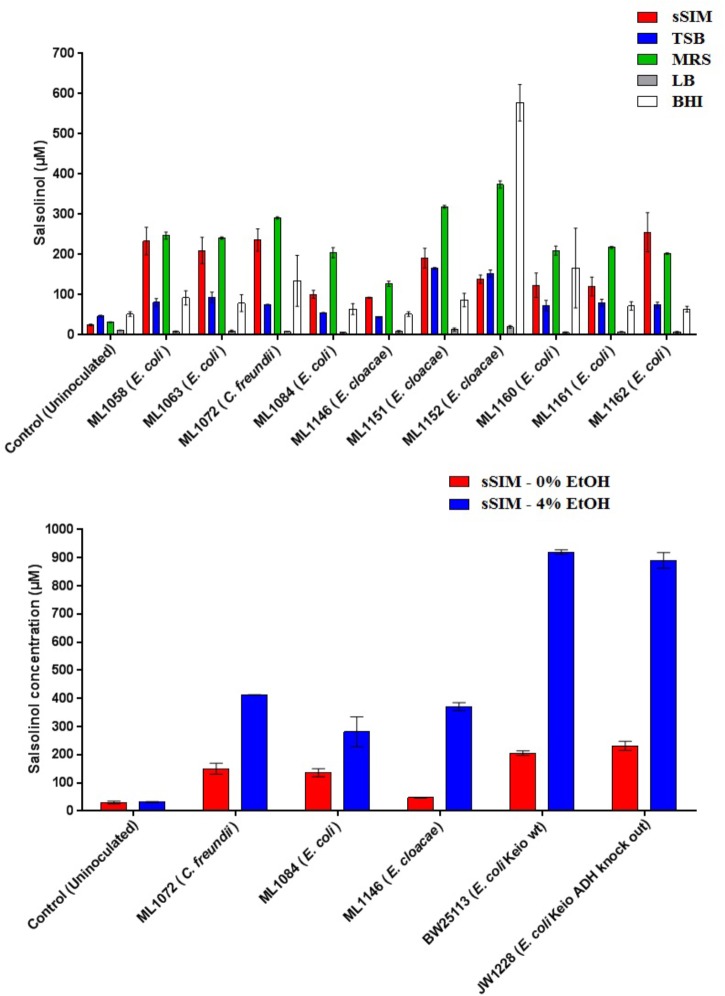
The formation of salsolinol by several species of *Enterobacteriaceae* is significantly influenced by the medium. **(Top)** Experiment comparing salsolinol production in four commonly used laboratory media in comparison to sSIM. **(Bottom)** Select organisms were grown in sSIM both with and without the addition of 4% ethanol. Each bar represents the mean ± S.E.M of quadruplicate cultures.

This study provides the first evidence that common and abundant members of the gut microbiota, namely *E. coli* and several related enterobacteria, have the capacity to produce salsolinol and that production is enhanced in the presence of alcohol (Figure 4). When *E. coli* is inoculated in a medium containing dopamine, distinct chromatographic evidence of the production of a new compound is evident (Figure 1). Isolation of this chemical through retention time based fractional collection yielded a sample suitable for mass spectrometry. As shown, the results of mass spectrometry, taken collectively, confirm the identification of the positive ion *m/z* 180 component in the isolated fraction as salsolinol (Figure 2). The data presented here are consistent with and fulfill the criteria for an endogenous metabolite to be considered an identified compound as set forth by groups including the Chemical Analysis Working Group of the Metabolomics Standards Initiative (Sumner et al., 2007) and others (Creek et al., 2014; Schymanski et al., 2014).

The identification of novel microbial metabolic activity by *E. coli* (the most highly studied biological organism in the world) that potentially impacts the understanding of the mechanisms by which neurodegeneration in the gut may influence the development of PD raises several questions. First, since *Enterobacteriaceae*, and *E. coli* in particular, are among the most highly studied biological organisms, why has the production of salsolinol not been described before either from actual *in vitro* experiments or from bioinformatic mining of the extensive databases that are now available? Most likely, the reason(s) is due to the environmental growth conditions in which the present work was conducted in that bacteria were evaluated in complex medium reflective of the host-based milieu (Villageliu et al., 2018). This was achieved through the simulated digestion of foodstuff in a manner consistent with host physiology (Mackie and Rigby, 2015). For many decades, LB broth has been the media most commonly used to grow *E. coli* (Paul De Vos, 2009). As shown in Figure 4, when grown in LB, salsolinol is not produced by *E. coli*. Presumably, the medium either lacks a fundamental co-factor or does not encourage the activation of requisite genes. That we found a large difference in the production of salsolinol across various media (Figure 4) demonstrates the value of growing organisms in conditions that closely mimic the digested food-containing *in vivo* milieu of the gut. As such, it should not therefore be surprising that no bioinformatics-based databases contain any indication that *E. coli* can produce the neurotoxin salsolinol.

**FIGURE 5:**
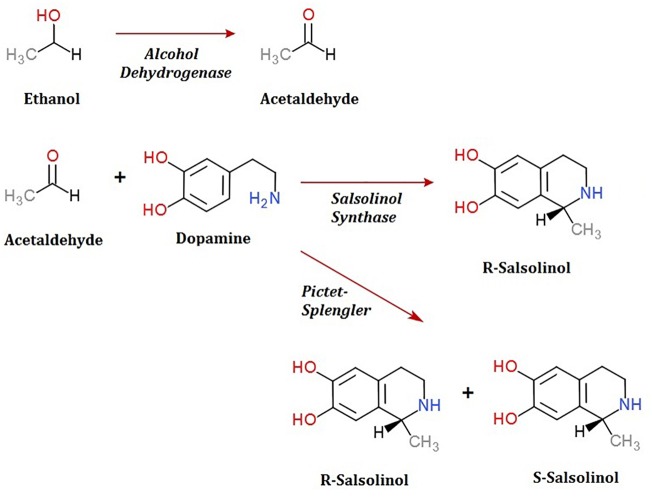
Microbial production of acetaldehyde from ethanol by alcohol dehydrogenase could provide acetaldehyde by either an enzymatic or non-enzymatic mechanism of salsolinol synthesis.

A second question concerns the genetic mechanism(s) by which salsolinol is made. Previously, several pathways leading to the production of salsolinol have been suggested (Chen et al., 2011). We have expanded on this proposal in order to incorporate the finding that alcohol enhances the production of salsolinol in microbes (Figure 5). It is well established that alcohol is converted to acetaldehyde via alcohol dehydrogenase in *E. coli.* An increase in acetaldehyde concentration would, by Le Chatelier’s principle (Treptow, 1980), increase the production of salsolinol for either an enzymatic or non-enzymatic mechanism. While an enzyme driven synthesis seems plausible, the non-enzymatic Pictet-Spengler mechanism (Stockigt et al., 2011) cannot be ruled out. Via the Pictet-Spengler reaction, a microorganism could facilitate the formation of salsolinol by producing aldehyde in an environment that contains dopamine. A common pathway for the production of aldehyde is via alcohol dehydrogenase which generates aldehyde from alcohol. We found that the presence of additional alcohol (Figure 4) does indeed favor the production of salsolinol. This could be consistent with either an enzyme that uses aldehyde as a substrate or the Pictet-Spengler mechanism. It is important to note however, that a single alcohol dehydrogenase mutant JW1228 from the Keio knock out collection (Baba et al., 2006) showed no statistical difference in salsolinol production in comparison to the wild type alcohol dehydrogenase mutant. This finding argues against mechanisms that rely on aldehyde. It is conceivable that an enzyme catalyzed reaction could form salsolinol in the absence of aldehyde, the only known salsolinol synthase enzyme forms salsolinol by a reaction with pyruvic acid, not aldehyde (Chen et al., 2018). Perhaps alcohol influences the microbial production of salsolinol by some other less direct pathway. It seems unlikely that aldehyde would be made by microorganisms in media like MRS, TSB, and BHI but not in LB. However, Pictet-Spengler mechanism still cannot be completely ruled out because the parent strain for this species is also known to have two copies of the alcohol dehydrogenase gene and it remains conceivable that one knock out is not sufficient to see a difference. Ultimately, mechanistic determinations will rely on follow up work.

As a putative salsolinol synthase has recently been described (Chen et al., 2018), we conducted a tblastn against the *E. coli* genome. Using the relatively loose parameters of BLOSUM45 and extension gap cost of 1, we were unable to find any homologous sequences. If there is a microbial enzyme, it is likely structurally distinct from the mammalian enzyme. We are currently conducting further research including the use of transposon mutagenesis to identify the genetic mechanism(s) by which *E. coli* produces salsolinol.

A third question concerns the conditions tested herein *in vitro* and whether they are representative of the conditions likely to be found *in vivo*. We tested what may seem like a relatively high exposure of 1 mM of dopamine and allowed the organisms twenty-four hours to interact with dopamine. Consider however, that within the localized pockets where microbes reside, this concentration could still be feasible. Dopamine is readily available and secreted throughout certain portions of the GI tract (Mezey et al., 1996, 1998) and a biofilm living in close proximity to dopamine secreting cells will experience a much higher effective concentration than cells which experience only the distant diffused concentration of the molecule. Research into the effects of dopamine on neural cell lines, including astroglial cells has covered the range of 1 mM (Hirrlinger et al., 2002) and some research has even tested concentrations as high as 100 mM (Chase et al., 2004).

The demonstration that one of the most abundant members of the gut microbiota, *E. coli*, can facilitate the synthesis of salsolinol does not by itself prove a causative link between the microbiota and PD. However, it is worth noting that a gut-based origin for PD has already been proposed in which dysregulation of the neuro-immune brain-gut axis could lead to the occurrence of enteric neuro-inflammatory conditions (Lionnet et al., 2018; Pellegrini et al., 2018). Interestingly, PD is frequently associated with functional gastrointestinal disorders including infrequent bowel movements, abdominal distension and constipation which can occur throughout all stages of the neurodegenerative process. Emerging evidence has suggested that there is an association between PD and an altered gut-microbiome, metabolites produced in the gut may modulate the disease. As one of the most investigated chemicals implicated in the development of PD is salsolinol (Kurnik-Łucka et al., 2018), it follows that the production of salsolinol by a gut microbe could prove highly relevant.

To date the role of salsolinol has been brain-centric (Kurnik-Łucka et al., 2018) with little or no role for its ability to effect neurodegeneration described outside of the central nervous system. Further, the demonstration that alcohol dramatically increases the production of salsolinol (Figure 4) suggests a possible mechanism by which to explore the purported association between excessive alcohol consumption and PD (Liu et al., 2013; Bettiol et al., 2015). It should be noted, however, that by itself this study does not offer sufficient evidence to establish that salsolinol production contributes to the pathogenesis of neurodegenerative diseases such as PD. However, in light of previous reports showing a correlation between salsolinol and various conditions (Kurnik-Łucka et al., 2018), the potential capacity for the microbiota to produce salsolinol *in vivo* should be considered. Further, previous reports which have measured salsolinol production in alcoholics have done so using urine (Matsubara et al., 1985; Dostert et al., 1991). Subsequent studies have produced conflicting results (Feest et al., 1992; Haber et al., 1996). The results of the present study suggest that fecal matter in addition to urine should be utilized in such studies.

In summary, this is the first known report of a microbe producing salsolinol. While dietary and host derived sources of salsolinol may still be relevant, the capacity for certain members of the microbiota to produce significant quantities of salsolinol suggests that they may be a source for much of the salsolinol that has been reported in host tissues. Follow up studies may use this as a basis upon which to examine the role of microbiota in the gut and potentially on the pathogenesis of disease. Further, this report may be viewed as complementary to that of the Braak hypothesis (Braak et al., 2003a,b, 2006) since it is conceivable that salsolinol produced within the gut could effect changes locally in the enteric nervous system. Though the total salsolinol burden experienced by a host might be manageable in a healthy host, it is possible that in some individuals a dysregulated microbiome or other pre-existing condition may facilitate the initiation of a disease cascade.

## Author Contributions

All authors contributed to the intellectual development of this paper. DV under the principal investigator ML made the initial observation and isolation of a unique metabolite derived from microbial activity. Characterization of this molecule was done in collaboration with the analytic expertise of DB. Follow up experiments were planned and conducted by both ML and DV.

## Conflict of Interest Statement

The authors declare that the research was conducted in the absence of any commercial or financial relationships that could be construed as a potential conflict of interest.

## Footnotes

1 mzcloud.org
